# Reply to: Rectifying misinformation on the climate intervention potential of ocean afforestation

**DOI:** 10.1038/s41467-024-47135-5

**Published:** 2024-04-09

**Authors:** Lennart T. Bach, Veronica Tamsitt, Jim Gower, Catriona L. Hurd, John A. Raven, Wouter Visch, Philip W. Boyd

**Affiliations:** 1 Institute for Marine and Antarctic Studies, University of Tasmania, Hobart, TAS Australia; 2 College of Marine Science, University of South Florida, St Petersberg, FL USA; 3 Fisheries and Oceans Canada, North Saanich, BC Canada; 4 Division of Plant Sciences, University of Dundee at the James Hutton Institute, Dundee, UK; 5 Climate Change Cluster, University of Technology, Sydney, NSW Australia; 6 School of Biological Sciences, University of Western Australia, Crawley, WA Australia

**Keywords:** Carbon cycle, Ocean sciences

**replying to** V. Smetacek et al. *Nature Communications* 10.1038/s41467-024-47134-6 (2024)

We thank Smetacek et al.^1^ for commenting on our study. Here, we provide our Reply to their third (modified) version of their Comment, noting that their first two versions made some substantially different arguments. Since responding to their first two versions provided potentially useful clarifications, we deposit our respective replies to those earlier versions in a data repository^2^. We encourage Smetacek et al. to also deposit their first two versions in a repository for full transparency and that the science community benefits from reading the full exchange of arguments.

In the revised version of their appeal, Smetacek et al. argue for the large potential of Ocean Afforestation while providing hypothetical solutions for its anticipated limitations^1^. For example: The reduction of the Ocean Afforestation potential through the nutrient re-allocation problem^3,4^? Can be fixed when extracting and recycling nutrients from seaweed biomass somehow^1^. Or: Restricted seaweed growth through nutrient limitation? Can be fixed through artificial upwelling where the upwelling pipes are built from seaweed material^1^. It is easy to come up with such hypothetical solutions, but much harder to pass them through a reality check.

The absence of such a reality check was what motivated us to utilize the “Great Atlantic Sargassum Belt (GASB)” as a natural analog for the scientific assessment of Ocean Afforestation^4^. Smetacek et al’s main criticism is that the GASB is not a suitable analog for Ocean Afforestation^1^. They provide mainly two arguments. First, the GASB is only a megatonne-scale phenomenon and hence fixes much too little CO_2_ to provide relevant insights for gigatonne-scale Ocean Afforestation. Second, *Sargassum* grows too slowly in the GASB to be comparable to necessarily fast-growing seaweeds envisioned for Ocean Afforestation.

Their scale argument is not reasonable. Ocean Afforestation would not go from zero to gigatonnes in one big step but this process would occur gradually with a certain rate of development^5^, as we can observe in sectors where seaweed is grown for products^6^. Thus, Ocean Afforestation would need to eventually pass the megatonne-scale of the GASB. If Ocean Afforestation could not provide verifiable CO_2_ removal at the megatonne-scale, then there would be little incentive to move forward to the gigatonne scale. Paradoxically, Smetacek et al. do not consider their scale argument when they advocate for the smaller “golden tide” events in the Yellow/East China Sea as more appropriate natural analog^1^.

Their dismissal of the GASB due to limited *Sargassum* growth rates is based on the argument that Ocean Afforestation depends on artificial upwelling and ocean iron fertilization to enable gigatonne-scale seaweed biomass production^1^. Indeed, already pioneering field trials in the 1970s found that the benthic seaweed *Macrocystis* does not grow in the open ocean unless fertilized with both nutrients sourced from depth and iron fertilizer sourced from land^7^. However, and in contrast to Smetacek et al.’s conclusion, such “irrigation” with nutrients does not solve the biogeochemical constraints of Ocean Afforestation as argued in the following.

The nutrient re-allocation problem discussed in our and other studies assumes: Nutrients that are used to fuel seaweed CO_2_ fixation are no longer available for phytoplankton^3,4,8,9^. Thus, the enhancement of an anthropogenic CO_2_ sink (Ocean Afforestation), would reduce a natural one (phytoplankton). As such, the net gain in CO_2_ fixation depends on how much more carbon the seaweeds can fix with the available amount of limiting nutrients than the phytoplankton were able to fix before their replacement^4,8^. This can be estimated from the difference in carbon-to-nitrogen (C:N) stoichiometry between seaweeds and phytoplankton, which is usually in favor of seaweeds^8^. However, in contrast to implications made by Smetacek et al.^1^, evidence suggests that nutrient-replete conditions (as they would be induced through artificial upwelling) will lower carbon-to-nutrient ratios in seaweed^8,10^, while potentially increasing phytoplankton carbon-to-nutrient ratios^11^. Even the one quantitative piece of evidence they provide for high C:N of golden tides supports this, as Smetacek et al. only cite the highest C:N (45 mol:mol) that occurred under nitrogen limitation, not the much lower value of 7.3 mol:mol the same study reported for arguably more nutrient replete golden tides closer to shore^12^. In other words, artificial upwelling could increase seaweed growth but would make Ocean Afforestation less efficient.

Smetacek et al.^1^ argue further that the nutrient re-allocation problem would not apply if nutrients were sourced via artificial upwelling from depth because the deep-ocean nutrient inventory is huge. This thought is conceptually misleading because what matters is not the pool size but what would happen to the artificially upwelled nutrients in the absence of artificial upwelling. For example, if nutrients were artificially upwelled from 530 m depth in the (sub)tropical North Atlantic, then they are available to fuel new CO_2_ fixation instantaneously. In the absence of such artificial upwelling these nutrients would have been upwelled naturally within the next 50 years^13^ and fuel phytoplankton productivity in the future. The deep ocean is not a homogenous reservoir but stratified itself and exchange between layers is limited^13^. It cannot simply be assumed that nutrients taken from some ocean depth through artificial upwelling would be fully or partially replaced through the remaining deep ocean nutrient pool. Thus, the nutrient re-allocation problem remains and must be integrated over timescales of which the deep ocean naturally exchanges nutrients with the surface ocean. This will make accounting for the nutrient re-allocation problem even more difficult.

Smetacek et al. argue that the calcification discount to Ocean Afforestation would not apply to “optimal conditions in Aquafarms,” due to fast growth of healthy seaweeds which outpace the growth if epibiont calcifiers and because seaweeds are able to defend themselves against epibionts with chemicals. However, it is well established that the onset and progression of biofouling in aquafarms depends on the life cycle of the epibiont, not primarily its growth or growth of the corresponding seaweed^14^. As such, the widespread practice to avoid biofouling by epibionts such as calcifying bryozoans is to avoid seaweed culturing during phases of epibiont spawning^15^. Thus, seaweed aquafarms for Ocean Afforestation would either need to accept a calcification discount or adjust (reduce) growth periods. Furthermore, macroalgae have limited antifouling properties against calcifying epibionts. Since brown algae contain phenolic defense compounds, such as phlorotannins, in discrete vesicles (physodes), they function mainly to deter grazing, not fouling^16^. Evidence suggests that larvae of epiphytic bryozoans preferentially settle on the meristematic growth part of the thallus and continue to grow on the macroalga’s tissue until erosion occurs^17^. While some macroalgae shed their epidermis to reduce epiphytes, its efficacy against calcifying epibionts remains limited^18^. Ultimately, fouling organisms impact the annual productivity of macroalgal farms worldwide, severely limiting the growth of macroalgae^19^.

To conclude: We note that Smetacek et al. provide a supportive narrative for Ocean Afforestation^1^. While our study revealed that Ocean Afforestation could potentially lead to additional CO_2_ sequestration in the oceans, we cautioned that it is associated with feedbacks that largely reduce the effectiveness of CO_2_ removal and impede quantification of its net climatic benefit^4^. The current exchange with Smetacek et al. has not altered this conclusion.

## Data Availability

The manuscript contains no new data or code.
